# Health Equity in Systematic Reviews: A Tutorial—Part 2 Implementing Health Equity Throughout Your Methods

**DOI:** 10.1002/cesm.70054

**Published:** 2025-10-15

**Authors:** Jennifer Petkovic, Jordi Pardo Pardo, Vivian Welch, Omar Dewidar, Lara J. Maxwell, Andrea Darzi, Tamara Lotfi, Lawrence Mbuagbaw, Kevin Pottie, Peter Tugwell

**Affiliations:** ^1^ University of Ottawa Ottawa Ontario Canada; ^2^ Bruyère Health Research Institute Ottawa Ontario Canada; ^3^ Temerty Faculty of Medicine University of Toronto Toronto Ontario Canada; ^4^ Department of Health Research Methods, Evidence & Impact McMaster University Hamilton Ontario Canada; ^5^ Department of Family Medicine Dalhousie University Halifax Nova Scotia Canada; ^6^ Department of Medicine University of Ottawa Ottawa Ontario Canada; ^7^ Methodological and Implementation Research Ottawa Hospital Research Institute Ottawa Ontario Canada; ^8^ School of Epidemiology and Public Health University of Ottawa Ottawa Ontario Canada

## Abstract

This is the second and final tutorial in a series on health equity. It provides detailed guidance for considering health equity in systematic reviews of interventions. We will explain how to include and report health equity in all remaining sections of the review.

## 1 How to Consider Health Equity in Systematic Reviews

The first tutorial in this series introduces health equity and provides advice on operationalizing the concept of health equity in your review. Please read the first tutorial before implementing the suggestions provided in this tutorial.

When planning a review, there are six main ways to consider health equity. We covered engaging interest‐holders and question formulation in the first tutorial. This tutorial will cover:

- Deciding what methods will be used to identify, extract, and appraise evidence related to health equity, including specific populations.
- Describing populations using health equity characteristics.
- Considering implications for “Summary of findings” tables (e.g., separate tables for disadvantaged populations, separate rows for differences in risk of events).
- Interpreting findings related to health equity in the discussion.

## 2 Objectives

If a review aims to assess health equity as an objective, authors should ensure that this is stated explicitly.

**Example.** A Cochrane review included a secondary objective to assess the effects of social media interventions on the health of populations who experience health inequity [1].

## 3 Methods Section

### 3.1 Getting Started: Decide What Methods Will be Used to Identify, Extract, and Appraise Evidence Related to Health Equity and Specific Populations

In the health equity‐related section in the methods, authors should describe how the review applies a health equity lens throughout, i.e. the methods used to identify evidence related to health equity and specific populations and settings. Authors should consider which population characteristics are of interest for the problem, condition, or intervention under review, as described in part 1.

Authors should consider the types of populations they expect to include in the review. Authors can consider using a table, such as the one suggested by the PRO‐EDI initiative and adapted by Cochrane to report the populations to be included in the review (https://www.trialforge.org/trial-diversity/pro-edi/) (Table 1) [2, 3].

**TABLE 1:** Adapted PRO‐EDI table.

Characteristic	Inclusion criteria of review (people we expect to see)	Representation in included studies (people who took part)
Age		
Sex/Gender		
Location (country/countries of data collection and site coordination)		
Optional characteristics:		
Race/ethnicity/ancestry		
Socioeconomic status		
Level of education		
Disability		
Sexual orientation		
Any other factors relevant for the review		

Authors should also describe how they will extract information to inform the characteristics of included studies and results sections and plan to report on population characteristics.

**Example.** The equity‐focused review of corticosteroids for COVID reported that they would extract participant characteristics data, including age, sex, and ethnicity as well as co‐morbidities. They also reported that they extracted equity‐related considerations, such as place of residence, occupation, religion, education, socioeconomic status, and social capital. They also reported that they planned to conduct subgroup analyses based on participant characteristics that may stratify the outcomes, including sex, age (< 70 years compared to 70 years and older), ethnicity, and place of residence (high vs. low‐ and middle‐income countries) [4].

Authors can consider reporting on health equity methods to appraise evidence related to health equity and specific populations and describe whether there are differences in the lived experience of these populations (e.g., racism, ageism, stigma, acceptability, other underlying determinants of health).

Authors should also describe the rationale for any methodological decisions related to specific populations and settings within the appropriate methods section, such as eligibility criteria, subgroup analyses, choice of outcomes, and databases chosen to locate studies. Box 1.

**Example.** A review of family‐centered interventions for Indigenous early childhood well‐being reported that their rationale for including non‐randomized study designs for many equity‐relevant reasons, including the inherent ethical considerations for working with Indigenous populations as well as the barriers that may affect participants of randomized trials, such as trials no incorporating Indigenous knowledge systems. The authors also planned to organize the data by Indigenous population and child′s age (although they were unable given the small number of included studies) [5].

Box 1 **What if the data or resources aren′t available?**

**Table:** 

Authors are always encouraged to contact the primary study authors for more information, if needed [24]. Review authors can state explicitly what they would have liked to explore but were unable to because of a lack of data in the studies or because of a lack of resources. This will help inform future research and updates of the review.

## 4 Results Section

### 4.1 Getting Started: Describe Health Equity Characteristics for Populations in Included Studies

Authors should report the characteristics of the populations considered. These population details can be summarized across included studies, including whether there are differences in baseline risk or prevalence of the problem or condition.

Authors can also assess and report whether the eligibility criteria of included studies were restricted to one or more population groups, intentionally excluding particular groups for which health equity data would have been relevant (as described in part 1). Using the table developed for the protocol (above), authors can complete the third column to report the characteristics of the populations included in the studies of the review. This allows authors to compare how the studies reflect the expected population characteristics. It may help authors judge the applicability of the evidence. It is important to note that even if the studies have not included all the population characteristics expected, the results may still be applicable to those who were not included in the studies of the review. Box 2.

**Example.** Tables 2 and 3‐ Summary of the characteristics of participants we would expect to see in the evidence and the actual participant characteristics extracted from the included studies (Using a review of interventions to prevent falls [6] and Systemic corticosteroids for the treatment of COVID‐19) [4].

**TABLE 2:** Example 1—completed PRO‐EDI table.

Example 1: Psychological and educational interventions for preventing falls in older people living in the community*Interventions: cognitive behavioral therapy, motivational interviewing, other psychological interventions, education, or combined psychological and education interventions*		
Characteristic	Inclusion criteria of review (people we expect to see)	Representation in included studies (people who took part)
Age	People aged 60 years or older	Studies of interventions to prevent falls included older adults, with mean ages over 70. This aligns with our expectations for whom these types of interventions would target (falls are more prevalent among older adults).
Sex/Gender	All sexes, all genders in studies about fall prevention.	The majority of participants were women, usually over 50% of participants and often over 70%. This aligns with our expectations.
Location (country/countries of data collection and site coordination)	Any country, urban or rural settings.	Most studies were from high income countries. The specific settings were often urban therefore we are not sure of the applicability of the evidence to nonurban settings where access to these types of interventions may differ.

**TABLE 3:** Example 2—completed PRO‐EDI table.

Example 2: Systemic corticosteroids for the treatment of COVID‐19		
Characteristic	Inclusion criteria of review (people we expect to see)	Representation in included studies (people who took part)
Age	Adults	Studies included adults with mean ages ranging from 54 to 73 years. This aligns with the target population.
Sex/Gender	All sexes, all genders in studies about COVID‐19	Women made up between 20% and 52% of the included study populations. This indicates that some studies included fewer than expected women
Location (country/countries of data collection and site coordination)	Any country, urban or rural settings.	Studies were conducted in high‐ and middle‐income countries but there were none conducted in low‐income countries. This means the results may not be applicable to other settings where there may be resource constraints or differences in protocols and standard care.
In the results section, authors should provide a brief overview of the health equity assessments, for example, reporting on any differences related to population characteristics.		

In the results section, authors should provide a brief overview of the health equity assessments, for example, reporting on any differences related to population characteristics.

**Example.** The rotavirus vaccine review reported the results according to mortality of the countries in which the studies were conducted: low‐, medium‐, or high‐mortality [7]. They reported, for example, that for Rotarix vaccine there was a 93% reduction in severe rotavirus diarrhea cases in low‐mortality countries, 79% reduction in medium‐mortality countries, and 58% reduction in high‐mortality countries.

Box 2 **You′re already reporting on most of this information.**

**Table:** 

In your results section, you are already reporting on the characteristics of the included studies. We are asking you to consider reporting on a few additional characteristics, such as in the above examples, and combining them into a summary table so that your readers can understand the population in all the studies.
If you are unable to report this information, you can explicitly state what information you couldn′t report to help inform future research and updates of the review.

### 4.2 Appraise the Included Studies

Authors should follow the recommended critical appraisal methods in the Cochrane Handbook, Chapters 8 and 25 [8, 9].

Specifically, for health equity, authors may appraise their included studies by considering whether the selection of participants, study designs and outcomes, and analysis strategy are appropriate and assess the potential influence of the context/setting and the process of the intervention. Authors should consider who the studies were conducted for and by, and assess study design factors to consider whether recruitment and attrition may influence health equity [10] or whether certain populations were excluded from the included studies [11].

One appraisal approach involves calculating the participant‐prevalence ratio (PPR) which can quantify the participation of specific populations in a study [12]. The PPR involves dividing the percentage of a subpopulation in the study by percentage of the subpopulation with the condition of interest. The PPR can also help to inform the generalizability of the evidence and assess the impact of eligibility criteria on the recruitment of these specified populations. Authors can report whether there are reasons for missing groups, for example, if recruitment attempts were made but only a small sample of the population subgroup was included or whether groups were purposefully excluded.

**Example.** An assessment of studies of extracorporeal cardiopulmonary resuscitation (ECPR) for cardiac arrest including data from four randomized controlled trials found substantial underrepresentation of women and black individuals [13]. The participant‐to‐prevalence ratio (PPR) for women was 0.48 (interquartile range [IQR]: 0.42–0.60), and for Black individuals it was 0.26 (IQR: 0.26–0.26), indicating significant disparities in trial recruitment relative to disease incidence. Notably, these disparities persisted even after adjusting for trial eligibility criteria, suggesting that the underrepresentation was not due to clinical or epidemiological exclusions, but may instead reflect broader health equity issues affecting the chain of survival.

### 4.3 Analysis of Health Equity‐Related Data

Health equity may be assessed through subgroup analyses or meta‐regression following the appropriate methods outlined in Chapter 10 of the Cochrane Handbook for Systematic Reviews of Interventions related to analyzing data and conducting meta‐analysis (https://www.cochrane.org/authors/handbooks-and-manuals/handbook/current/chapter-10#section-10-11). Any subgroup analysis should be conducted and interpreted with caution; they should be prespecified and justified to avoid spurious findings [14, 15, 16].

If subgroup or sensitivity analyses were planned related to health equity, these should be reported, or authors should state why they were not done.

**Example.** A re/view of interventions for smokeless tobacco cessation planned to conduct subgroup analyses by geographical/cultural origin. However, in the results section, the authors stated: “We were unable to conduct our planned subgroup analyses because of insufficient variety among the included studies in the geographical region…” [17].

## 5 Summary of Findings Tables

### 5.1 Getting Started: Consider Implications for “Summary of Findings” Tables (e.g., Separate Tables for Disadvantaged Populations, Separate Rows for Differences in Risk of Events)

Depending on the review, authors may choose to present health equity‐related findings in the Summary of Findings tables. If appropriate, authors can present separate tables to present different populations, for example, countries with low under‐5 mortality rates separately from high under‐5 mortality rates (e.g., below), which relate to regional differences and country income classifications. Alternatively, authors may choose to present different risks using separate rows within the same table. These decisions should be driven by evidence and the knowledge and perspectives of interest‐holders. See the Cochrane Handbook for Systematic Reviews of Interventions for information about templates (https://training.cochrane.org/handbook/current/chapter-14#section-14-1). Box 3.

**Example.** The rotavirus vaccine review presented separate Summary of Findings tables for low‐mortality, medium‐mortality, and high‐mortality countries based on country under‐5 mortality [7], https://www.cochranelibrary.com/cdsr/doi/10.1002/14651858.CD008521.pub6/full#CD008521‐sec‐0008).

Another review, assessing compression stockings for preventing deep vein thrombosis in airline passengers presents the evidence on separate rows for each outcome, according to the population risk (low‐risk or high‐risk) [18], https://www.cochranelibrary.com/cdsr/doi/10.1002/14651858.CD004002.pub4/full#CD004002‐sec‐0008).

Box 3 **Summary of findings formats.**

**Table:** 

Consider the Summary of Findings table format and whether presenting the information differently, using an alternative format, would help explain your results. If not, continue using the standard format.

## 6 Discussion Section

### 6.1 Getting Started: Interpret Findings Related to Health Equity in the Discussion

Authors should discuss the applicability of the results for different populations and settings, especially those specified in the design of the review. Additionally, reflections should be made on whether there were differences observed in the effectiveness of the intervention. Outcomes may be valued differently across populations, which can lead to varying perceptions of benefit and, ultimately, differential impacts of the intervention.

**Example.** The corticosteroids for COVID review reported that their included studies were from high‐income countries with only 12% from middle‐income countries. No studies were conducted in low‐income countries and therefore the authors report that the evidence may not be applicable in lower resource settings because of differences in standard care as well as other constraints, such as shortages of hospital beds, oxygen or other respiratory support [4].

In the review′s conclusions, authors should discuss the implications of their findings for health equity in research, practice and policy. This may include deriving research recommendations to address gaps in the evidence base, such as the absence of data for specific populations, or the need for targeted questions that can improve the certainty of evidence or inform equitable decision‐making (e.g., acceptability and feasibility). Given the importance of assessing applicability using metrics like the PPR, authors should also highlight the need for disaggregated prevalence and incidence data to support future assessments. This includes encouraging the collection and reporting of baseline information on population characteristics and social context, which are often missing or inconsistently reported in studies.

Biological differences across populations can also lead to avoidable disparities in health outcomes if not adequately addressed. For example, anatomical variations such as differences in carotid artery size between males and females may influence the effectiveness or safety of an intervention. An additional concern for health equity would invite reflection on how biological variation intersects with systemic bias. This has been illustrated in the case of cardiovascular disease in women where differences in symptom presentation have been historically under‐recognized due to gender bias in both clinical research and practice. Box 4.

**Example.** The schoolfeeding review reported that their evidence suggests that poorer and more undernourished children may be more responsive to supplementary feeding programs. Therefore, implications for practice include considering a targeted approach, if necessary, due to financial or other resource constraints [19].

Box 4 **One last reminder.**

**Table:** 

When writing your discussion, reporting that you were unable to explore possible health inequities is an important finding. This information can inform future studies and updates of the review.

## 7 Summary Checklist

1. Consider health equity when formulating the review question
  - Consider using a logic model.
2. Engage appropriate interest‐holders.
3. Decide and report on how you will identify, extract, and appraise evidence related to equity.
4. Describe the equity characteristics for the populations included in the studies of your review.
5. Consider how you will present equity‐related data, for example, adapting “Summary of findings” tables (e.g., separate tables for disadvantaged populations, separate rows for differences in risk of events).
  - Report all analyses related to equity (whether they could be conducted or not).
6. Include an interpretation of the findings related to health equity.

## 8 Reporting Health Equity

There are many tools for reporting on health equity within your review. For health equity‐focused reviews, authors can follow the PRISMA‐Equity Reporting Guideline to ensure the health equity is reported completely and transparently (Welch et al. 2012). For other reviews, authors can use the appropriate guideline from the following:

‐ The Sex and Gender Equity in Research (SAGER) guideline is for reporting of sex and gender in study design, data analyses, results and interpretation of findings [20].
‐ The Consolidated criteria for strengthening reporting of health research involving Indigenous peoples—the CONSIDER statement is a synthesis and prioritization of existing national and international statements and guidelines [21].
‐ JAMA has recommendations and suggestions to encourage fairness, equity, consistency, and clarity in use and reporting of race and ethnicity in medical and science journals [22] as well as the CMAJ guidance on reporting race and ethnicity in research [23].
‐ The PRO‐EDI website has guidance for how to report study characteristics and present the information in summary tables (https://www.trialforge.org/trial-diversity/pro-edi/).
‐ EPPI Centre guidance for considering and reporting methods for investigating health equity in systematic reviews and other evidence syntheses (https://eppi.ioe.ac.uk/cms/Default.aspx?tabid=3939).

### 8.1 Further Reading and Online Content

We have developed a micro‐learning module, including a short quiz with feedback, to illustrate the common errors covered in this tutorial (https://share.gomolearning.com/sharelink/f47d14406c77aceee0f9e3bc055df41167ec9066842ae04553/).

Additional material is also provided in the learning module “Health Equity in Systematic Reviews” developed by Cochrane Training. This can be found here: https://www.cochrane.org/learn/courses-and-resources/interactive-learning/module-11-health-equity-systematic-reviews.

## Author Contributions

**Jennifer Petkovic:** conceptualization, writing – original draft, writing – review and editing, methodology, project administration. **Jordi Pardo Pardo:** conceptualization, methodology, writing – review and editing. **Vivian Welch:** conceptualization, methodology, writing – review and editing. **Omar Dewidar:** writing – review and editing. **Lara J Maxwell:** writing – review and editing. **Andrea Darzi:** writing – review and editing. **Tamara Lotfi:** writing – review and editing. **Lawrence Mbuagbaw:** writing – review and editing. **Kevin Pottie:** writing – review and editing. **Peter Tugwell:** writing – review and editing.

## Ethics Statement

All authors are members of the leadership team of the Cochrane Health Equity Thematic Group.

## Consent

The authors have nothing to report.

## Conflicts of Interest

The authors declare no conflicts of interest.

## Peer Review

The peer review history for this article is available at https://www.webofscience.com/api/gateway/wos/peer-review/10.1002/cesm.70054.

## Supporting information

CESM+Declaration+of+Interest FORM.

## Data Availability

Data sharing not applicable to this article as no datasets were generated or analyzed during the current study.
